# The Effects of Physical Exercise on Depression and Anxiety in Cancer Patients: A Systematic Review

**DOI:** 10.3390/clinpract15100180

**Published:** 2025-09-28

**Authors:** Giacomo Farì, Annatonia Fai, Francesco Quarta, Morena Pitruzzella, Cristiano Sconza, Matteo Luigi Giuseppe Leoni, Giustino Varrassi, Marco Filipponi, Giuseppe Rollo, Alessio Baricich, Andrea Bernetti

**Affiliations:** 1 Department of Experimental Medicine (Di.Me.S), University of Salento, 73100 Lecce, Italy; giacomo.fari@unisalento.it (G.F.); andrea.bernetti@unisalento.it (A.B.); 2 Department of Translational Biomedicine and Neuroscience (DiBraiN), Aldo Moro University, 70121 Bari, Italy; 3 Department of Biological and Environmental Science and Technologies (Di.S.Te.B.A.), University of Salento, 73100 Lecce, Italy; francesco.quarta6@studenti.unisalento.it; 4 Rehabilitation Unit, Don Orione Hospital, 00183 Rome, Italy; morenapitru@gmail.com; 5 Rehabilitation Unit, IRCCS Humanitas Research Hospital, 20089 Milan, Italy; cristianosconza@gmail.com (C.S.); alessio.baricich@hunimed.eu (A.B.); 6 Department of Medical and Surgical Sciences and Translational Medicine, Sapienza University of Rome, 00189 Rome, Italy; matteolg.leoni@gmail.com; 7 Pain Medicine, Paolo Procacci Foundation, 00193 Rome, Italy; g.varrassi@fondazioneprocacci.org; 8 Department of Orthopaedics and Traumatology, Vito Fazzi Hospital, 73100 Lecce, Italy; filipponimarco@yahoo.it (M.F.); rollo.giuse@gmail.com (G.R.); 9 Department of Biomedical Sciences, Humanitas University, Pieve Emanuele, 20072 Milan, Italy; 10 Infradepartmental University Program of Physical and Rehabilitation Medicine, “V.Fazzi” Hospital, ASL Lecce, 73100 Lecce, Italy

**Keywords:** anxiety, cancer patients, people with cancer, cancer rehabilitation, depression, exercise therapy, medical oncology, mental health, physical exercise

## Abstract

**Background**: Depression affects around 280 million people globally and is one of the main causes of disability. Among people with cancer, depression and anxiety affect 20–25%, significantly reducing quality of life, adherence to treatments, and survival. Despite the availability of pharmacological and psychological treatments, their application can be limited by side effects, accessibility, and costs—especially in low- and middle-income countries. Physical exercise is emerging as a valuable complementary strategy, improving both physical and mental well-being. Nevertheless, structured exercise programs are still rarely implemented in oncology. This review aims to provide evidence-based recommendations for integrating physical activity into mental health support for people with cancer. **Methods**: This review includes six randomized controlled trials (RCTs) evaluating physical exercise interventions for depression and anxiety in people with cancer aged over 18 years. Included studies compared exercise interventions to control or standard care and reported outcomes related to psychological well-being and adverse effects. Exclusion criteria included non-original studies, non-English articles, and works not focused on exercise. The search was conducted in PubMed, Scopus, Google Scholar, and the Cochrane Library using MeSH terms and Boolean operators. The review protocol was registered in PROSPERO (CRD42025637522). **Results**: Exercise interventions—such as aerobic, resistance, and mind–body practices—proved effective and feasible across cancer types. Benefits were seen in both psychological and physical outcomes. However, variations in protocols and outcome measures, as well as a lack of long-term data, limit generalizability. **Conclusions**: Personalized exercise programs can significantly reduce depression and anxiety in people with cancer. Standardized core methods, long-term research, and systemic support are needed to integrate exercise into routine oncology care.

## 1. Introduction

Depression impacts nearly 280 million individuals across the globe and stands as one of the foremost causes of disability worldwide [1]. In 2019, it was responsible for more than 47 million disability-adjusted life years (DALYs), solidifying its position as the leading factor contributing to the global mental health burden [1]. Despite often being underestimated, depression is a profoundly disabling condition with serious implications for public health on a global scale.

A 2015 study published in *JAMA Psychiatry* revealed that individuals suffering from mental health disorders, including depression and anxiety, face twice the risk of mortality compared to the general population, with 67.3% of deaths attributed to natural causes and 17.5% to unnatural causes [2]. Worldwide, mental disorders are responsible for about 14.3% of all deaths, amounting to roughly 8 million fatalities annually, underscoring the urgent need for targeted and accessible interventions [2]. Symptoms of depression typically include persistent low mood, diminished interest, sleep problems, and suicidal ideation, whereas anxiety is marked by excessive worry and muscle tension [3]. These conditions significantly impair quality of life, particularly among individuals living with chronic diseases such as cancer [4].

In people diagnosed with cancer, depression and anxiety frequently coexist, with prevalence estimates ranging from 20% to 25% [5]. These mental health issues often emerge due to the profound psychological and physical toll of cancer diagnosis and treatment. Beyond diminishing quality of life, these disorders are linked to reduced treatment adherence, weakened immune responses, increased morbidity, and elevated mortality risk [6].

These multidimensional effects underscore the need for comprehensive strategies to manage depression and anxiety in this population.

Traditional treatment approaches, including pharmacological interventions and psychotherapy, are commonly used; however, they have limitations, particularly in oncology settings. Pharmacological treatments may interact with cancer therapies, leading to potential side effects or reduced efficacy, while access to psychotherapy may be constrained by logistical or financial barriers [7]. Consequently, there is growing interest in integrative and lifestyle-based interventions, including physical exercise, as adjunctive therapies.

Physical exercise has demonstrated robust benefits for both physical and mental health in diverse populations [8,9,10,11,12]. Specifically, in people with cancer, exercise has been shown to alleviate treatment-related side effects such as fatigue, nausea, and neuropathy while also improving cardiovascular fitness, strength, and overall functional capacity [13].

In the oncological context, exercise has been shown to improve physical symptoms related to treatments, such as fatigue, nausea, and neuropathy, as well as enhance cardiovascular and muscular capacity. Specifically, in patients with breast cancer undergoing chemotherapy, physical activity has demonstrated positive effects in reducing cancer-related cognitive impairment (CRCI), which can affect attention, memory, and executive function in up to 80% of patients [14].

In people with cancer, regular physical exercise has been associated with reduced fatigue, enhanced self-esteem, and improved coping strategies, all of which contribute to better mental health outcomes [15,16,17].

However, the systematic integration of structured exercise programs into oncology care is still limited. Barriers include patients’ physical conditions, lack of awareness among healthcare providers, and the scarcity of dedicated resources.

This article aims to examine the current evidence on the effectiveness of physical exercise in the treatment of depression and anxiety in people with cancer. The goal is to provide practical recommendations for the implementation of physical activity as an adjunctive therapy in daily clinical practice, thus offering a holistic and sustainable approach to improving psychological well-being in this vulnerable population.

## 2. Materials and Methods

### 2.1. Data Sources, Search Strategy and Study Selection

This review was conducted in accordance with the Preferred Reporting Items for Systematic Reviews and Meta-Analyses (PRISMA) guidelines [18] (The PRISMA 2020 checklist is provided in the Supplementary Materials). The protocol was prospectively registered in PROSPERO (n◦ CRD42025637522). A comprehensive literature search was performed across the PubMed, Google Scholar, Scopus, and Cochrane Library databases, utilizing Medical Subject Headings (MeSH) terms where appropriate. The eligible studies were identified by using the following Boolean search syntax: ((“cancer patients” OR “patients with cancer” OR “adults” OR “oncology patients” OR “breast cancer patients” OR “lung cancer patients” OR “colorectal cancer patients” OR “cancer survivors”) AND (“physical exercise” OR “exercise therapy” OR “exercise programs” OR “exercise intervention”) AND (“control group” OR “non-exercise group” OR “standard care” OR “usual care” OR “no intervention” OR “placebo” OR “non-exercising participants” OR “comparator group”) AND (“anxiety” OR “depression” OR “mental health” OR “mental health” OR “psychological well-being”)). Subsequently, the following filters were applied—text availability: full text; species: humans; languages: English. The search syntax used for the PubMed database was a combination of MeSH database and Boolean search syntax: (“Neoplasms” [MeSH Terms] OR “cancer patients” OR “patients with cancer” OR “oncology patients” OR “breast cancer patients” OR “lung cancer patients” OR “colorectal cancer patients” OR “cancer survivors” [MeSH Terms] OR “cancer survivors”) AND (“Exercise” [MeSH Terms] OR “Exercise Therapy” [MeSH Terms] OR “physical exercise” OR “exercise intervention” OR “exercise programs”) AND (“Control Groups” [MeSH Terms] OR “control group” OR “non-exercise group” OR “standard care” OR “usual care” OR “no intervention” OR “placebo” OR “non-exercising participants” OR “comparator group”) AND (“Depression” [MeSH Terms] OR “Anxiety” [MeSH Terms] OR “Mental Health” [MeSH Terms] OR “psychological well-being” OR “depression” OR “anxiety” OR “mental health”); for Google Scholar: (“cancer patients” OR “oncology patients” OR “breast cancer” OR “lung cancer” OR “colorectal cancer” OR “cancer survivors”) AND (“physical exercise” OR “exercise therapy” OR “exercise intervention” OR “exercise program”) AND (“depression” OR “anxiety” OR “mental health” OR “psychological well-being”); for Scopus: TITLE-ABS-KEY ((“cancer patients” OR “oncology patients” OR “breast cancer” OR “lung cancer” OR “colorectal cancer” OR “cancer survivors”) AND (“physical exercise” OR “exercise therapy” OR “exercise intervention” OR “exercise program*”) AND (“depression” OR “anxiety” OR “mental health” OR “psychological well-being”) AND (“control group” OR “usual care” OR “comparator group” OR “no intervention” OR “placebo”)); for Cochrane Library: (cancer patients OR oncology patients OR breast cancer OR lung cancer OR colorectal cancer OR cancer survivors) AND (physical exercise OR exercise therapy OR exercise intervention OR exercise program*) AND (control group OR standard care OR usual care OR comparator OR placebo OR no intervention) AND (depression OR anxiety OR mental health OR psychological well-being). To identify additional relevant publications, the reference lists of the selected articles were manually screened. The literature search was conducted between 17 December 2024 and 3 January 2025. Potentially relevant articles were screened and selected based on predefined inclusion and exclusion criteria.

Inclusion criteria were as follows: (1) oncology patients aged over 18 years, (2) Randomized Controlled Trials (RCTs), (3) studies that include assessments of depression and anxiety levels.

Exclusion criteria concerned studies such as comments, expert opinions, case reports, case series, conference meeting abstracts, surveys, reviews, editorials, systematic reviews, meta-analyses, letters and articles not focusing on physical exercise interventions.

### 2.2. Data Extraction and Outcome Measures

Four investigators independently screened titles, abstracts, and full-text articles to determine study eligibility. Any disagreements were resolved by consensus with three additional experienced investigators. Data extraction was performed using standardized forms, capturing details such as the type of intervention and population characteristics.

### 2.3. Quality Assessment

The included studies were evaluated qualitatively using the Modified Oxford Quality Scoring System, which is also referred to as the Modified Jadad Score [19]. This tool consists of a four-item scale assessing key methodological aspects such as randomization and concealment of treatment allocation groups, withdrawals and dropout rates, adherence to inclusion and exclusion criteria, and clarity in describing statistical methods reported in the studies. Each criterion was assessed independently by the four investigators mentioned earlier. The Modified Jadad score ranges from 0 to 5, and each question has a dichotomous answer (yes—1 point; no—0 points) [20]. A higher score indicates better study quality. If a study had a modified Jadad score greater than 3 points, it was considered high quality; if the score was 2–3 points, it was considered moderate quality; and if the score was less than 2 points, it was considered low quality. As stated by Olivo et al. [19], the Modified Jadad Score demonstrated the best evidence for validity and reliability in quality assessment in this field. However, there is still a need to develop a valid and reliable scale specifically for assessing the methodological quality of trials.

### 2.4. Risk of Bias Assessment

The risk of bias in all included randomized controlled trials (RCTs) was evaluated according to the six domains outlined by the Cochrane Collaboration tool (Rob 2.0) [21]. These domains encompass: (1) selection bias, related to the methods of random sequence generation and allocation concealment; (2) performance bias, which considers the blinding of participants and personnel; (3) detection bias, concerning the blinding of outcome assessors; (4) attrition bias, addressing the completeness of outcome data; (5) reporting bias, involving selective outcome reporting; and (6) other biases, covering any additional sources of bias not addressed by the previous categories. Each domain was rated as “low risk” (green), “high risk” (red), or “unclear risk” (yellow). To summarize these assessments, the authors’ judgments were visually represented both as percentage distributions and aggregate summaries across all studies using the web-based tool “robvis” (Rob 2.0 version) [22].

## 3. Results

### 3.1. Identification of Study

**Figure 1:**
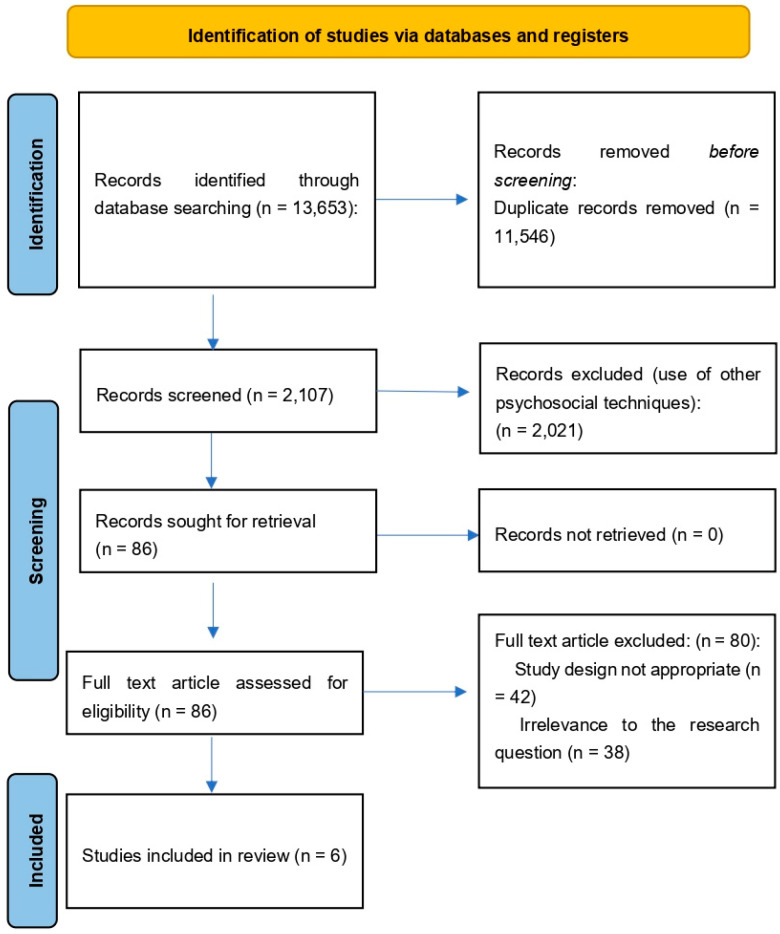
Study selection and eligibility screening flow according to PRISMA guidelines.

The studies were identified through a search of four databases (PubMed, Google Scholar, Scopus and Cochrane Library). At the end of the selection process, 13,653 articles were extracted. Duplicates were removed (n = 11,546). Thus, all remaining titles and abstracts were screened, and review articles, meta-analyses, case reports, letters, editorials, systematic reviews, cadaveric studies, conference abstracts, and studies lacking accuracy and safety evaluations were excluded. Subsequently, the full text of the remaining 86 articles was assessed for eligibility. Finally, 6 research articles were included in the review (Figure 1). 

### 3.2. Characteristics of the Included Studies

Table 1 shows the characteristics of the included studies retrieved from the systematic review. Sample size, a brief summary of the research design, the collected data, outcomes and results were analyzed for all six studies. The selected studies focused on the effects of physical exercise in patients with oncological diseases.

A total of six studies, including 509 patients, were analyzed in this review. These studies investigate the effects and potential benefits of physical exercise on depression and anxiety in people with cancer. All included studies evaluated the effectiveness of physical exercise through multi-week exercise programs, comparing intervention groups with control groups.

The body of evidence highlights the multifaceted benefits of exercise, encompassing physical, psychological, and functional domains while also addressing disease-specific challenges and comorbidities. From aerobic and resistance training to mind–body practices such as Tai Chi, the versatility of exercise interventions is emphasized, showcasing their adaptability to diverse patient populations.

Aydin et al. (2021) [23] provided compelling evidence of the dual benefits of exercise in people with breast cancer, demonstrating improvements in QoL alongside significant reductions in depression and anxiety levels. This study underscores the critical interplay between physical exercise and psychological health, suggesting that tailored exercise regimens can simultaneously address the physical and emotional toll of cancer. Similarly, Charati et al. (2022) [28] explored the efficacy of exercise in enhancing shoulder function while alleviating symptoms of depression and anxiety in women with breast cancer. Their findings highlight the potential for exercise to target localized impairments while offering broader mental health benefits, thereby addressing both physical and psychological recovery needs.

The role of exercise in managing depression and anxiety among older cancer patients has been further validated by Mikkelsen et al. (2022) [24], who demonstrated that structured interventions can be safely implemented in populations traditionally considered at higher risk for exercise-related complications. This study strengthens the argument for personalized exercise regimens that account for individual health conditions, physical capabilities, and age-related limitations. Such an approach ensures that the benefits of exercise are accessible to a broader spectrum of people with cancer, including those with advanced age or significant comorbidities.

Focusing on lung cancer survivors, Cavalheri et al. (2017) [25] highlighted the benefits of exercise during the recovery phase post-treatment. Their findings indicated significant enhancements in physical fitness and QoL but no evidence of significant changes in depression and anxiety.

Dieli-Conwright et al. (2018) [26] demonstrated that exercise interventions tailored to overweight and obese breast cancer survivors resulted in improvements in emotional well-being, physical fitness, bone health, and quality of life. Collectively, these studies highlight the potential of exercise to address cancer-specific health challenges, such as mental health, osteoporosis, and weight management, while improving overall psychological and physical well-being.

Ho et al. (2020) [27] examined the combined effects of dietary and physical exercise interventions in colorectal cancer survivors, revealing significant benefits for health-related QoL, anxiety, and depression. Although this study involves a multimodal intervention, it was included to reflect the real-world application of lifestyle modifications in this population. The effects should be interpreted cautiously, as some benefits may be attributable to dietary changes rather than exercise alone. However, it suggests that the integration of exercise with other lifestyle modifications may produce synergistic benefits.

### 3.3. Assessment of Methodology and Quality of the Studies

The methodological quality of the included studies was assessed using the Modified Jadad Scale, as shown in Table 2. In this systematic review, two studies were identified as having high quality (scores > 4) [24,26], while four studies were categorized as having moderate quality (scores 3 to 4) [23,25,27,28].

### 3.4. Evaluation Risk of Bias

**Figure 2:**
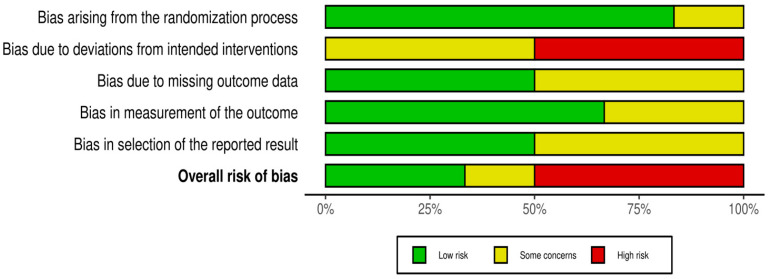
Risk of bias graph: review of the authors’ judgments about each risk of bias item presented as percentages across all the included studies.

The risk of bias graph is presented in Figure 2. The overall level of risk of bias in all of the studies selected in this systematic review revealed some concerns regarding the randomization process (selection bias), deviations from the intended interventions, missing outcome data, bias in measurement of the outcome and selective reporting of results. More specifically, 33.3% of the studies showed a low risk of bias, 16.7% showed some concerns, and 50% showed a high risk of bias.

**Figure 3:**
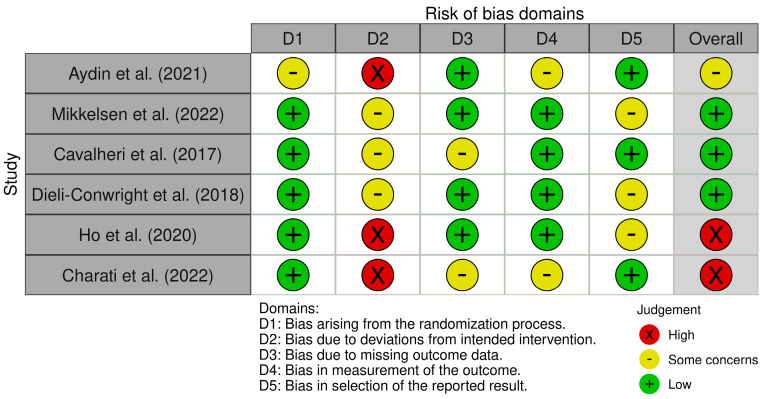
Risk of bias summary: review of the authors’ judgements [23,24,25,26,27,28] about each risk of bias item for each included study.

A risk of bias summary is reported in Figure 3. It reveals that three out of six studies had a low risk of bias [24,25,26]. Conversely, two of the six studies showed a high risk of bias due to deviations from the intended intervention [27,28]. Moreover, four studies demonstrated a low risk of bias related to missing outcome data [23,24,26,27] and four studies showed a low risk of bias in the measurement of outcomes [24,25,26,27]. Lastly, three studies presented a low risk of bias in the selection of the reported results [23,25,28].

## 4. Discussion

In light of these considerations, the findings of this review confirm the significant role of physical exercise as a complementary intervention in managing psychological symptoms, particularly depression and anxiety, in people with cancer. The studies examined provide a solid foundation to support the effectiveness of physical activity in diverse clinical contexts, but they also highlight certain critical issues that warrant further investigation.

Across these studies, several consistent themes emerge. First, exercise interventions are not only feasible but also effective across various cancer types, stages, and patient demographics. This universality underscores the adaptability of exercise as a therapeutic modality. However, the heterogeneity in exercise protocols—varying in intensity, duration, frequency, and type—highlights the need for greater standardization in future research. Establishing evidence-based guidelines that can be universally adopted will enhance the comparability of findings and facilitate their translation into clinical practice.

Furthermore, the reviewed studies consistently report short-term benefits, but the lack of long-term follow-up data limits our understanding of the sustainability of these improvements. Future research should prioritize longitudinal designs to evaluate whether exercise benefits persist over time and identify factors that influence adherence and effectiveness.

Another challenge lies in the variation in outcome measures, which complicates direct comparisons across studies. For example, while some studies, such as those by Dieli-Conwright et al. (2018) [26] and Cavalheri et al. (2017) [25], prioritize physical fitness metrics, others, like Aydin et al. (2021) [23] and Charati et al. (2022) [28], focus on psychological outcomes such as depression and anxiety. A more comprehensive approach that integrates physical, psychological, and QoL metrics is needed to capture the full spectrum of exercise-related benefits.

Despite the compelling evidence, significant barriers to exercise participation persist. These are especially relevant for vulnerable patient populations, where limitations in physical activity are common—particularly during times of healthcare system strain. For example, the recent COVID-19 pandemic significantly reduced access to physical exercise opportunities, particularly for frail individuals [29,30,31,32]. Among people with cancer specifically, factors such as cancer-related fatigue, limited access to exercise facilities, financial constraints, and psychological reluctance may further hinder participation.

Mikkelsen et al. (2022) [24] offer valuable insights into overcoming these barriers by tailoring interventions to individual capabilities and preferences. Strategies such as home-based exercise programs, telehealth platforms, and community partnerships can enhance accessibility and adherence [33,34].

However, broader implementation will require systemic changes at the policy level. Increased funding for exercise programs, integration of physical exercise into standard cancer care protocols, and training for healthcare providers to advocate for exercise are critical steps. Establishing exercise oncology as a recognized discipline within cancer care could further institutionalize the role of physical activity. Integrating personalized exercise programs into standard cancer care, supported by appropriate policies and multidisciplinary collaboration, could lead to significant improvements in managing depression and anxiety among people with cancer and survivors.

## 5. Conclusions

The studies reviewed highlight the transformative potential of exercise interventions in improving the quality of life and psychological well-being of people with cancer, with a particular focus on reducing depression and anxiety. Exercise programs, ranging from aerobic and resistance training to mind–body practices like Tai Chi, have demonstrated their effectiveness in enhancing psychological health across various cancer types, stages, and patient demographics. While the evidence is strong, significant challenges remain, such as the need for standardized exercise protocols, comprehensive outcome measures, and long-term follow-up data. Addressing barriers to participation, such as fatigue, access issues, and patient reluctance, is essential for the broader adoption of interventions.

Another limitation of this systematic review is the heterogeneity of intervention types, although all are related to physical exercise. Due to the heterogeneity of the analyzed studies and the limitations of this review, further high-quality research is needed to better explore and assess the potential of this new rehabilitative approach, in order to increase the supporting scientific evidence and allow for greater relevance within international guidelines.

Future research should focus on refining exercise guidelines, exploring the long-term psychological benefits, and leveraging multimodal interventions to maximize their impact on mental health outcomes. By adopting exercise as a core component of comprehensive cancer care, the healthcare community can make a substantial contribution to the psychological well-being and recovery of people with cancer.

## Figures and Tables

**Table 1:** Summary characteristics of the selected studies.

Title	Authors and Year of Publication	Sample	Research Design	Collected Data and Outcomes	Results
The Effect of Exercise on Life Quality and Depression Levels of Breast Cancer Patients	Aydin et al. (2021) [23]	48 women diagnosed with breast cancer who completed treatment.	This RCT divided the study population into two groups: - Intervention group: engaged in a structured 12-week exercise program. - Control group: Did not participate in any exercise intervention.	Depression levels were measured using the WHOQOL-BREF, EORTC-QLQ-C30 quality of life assessments and BDI, before and after the intervention.	Following the 12-week program, the intervention group demonstrated a significant reduction in depression levels. In contrast, no notable changes were observed in the control group. These findings suggest that a structured exercise program can effectively alleviate symptoms of depression in breast cancer survivors.
Exercise Intervention Among Older Patients with Advanced Cancer: Results from a Randomized Controlled Trial	Mikkelsen et al. (2022) [24]	The study involved 84 older adults (aged ≥65 years) diagnosed with advanced-stage (III/IV) pancreatic, biliary tract, or non-small cell lung cancer, all of whom were undergoing systemic oncological treatment.	RCT; a 12-week multimodal exercise intervention comprising the following components: - Twice-weekly supervised exercise sessions - A home-based walking program - Post-exercise protein supplementation - Nurse-led support and counseling	Symptoms of depression and anxiety plus physical function.	A 12-week multimodal exercise program with targeted support proved effective in enhancing physical function, endurance, muscle strength, overall physical activity level and mental well-being in older adults with advanced cancer undergoing oncological treatment. Participants in the intervention group also experienced reductions in symptoms of depression and anxiety.
Exercise training for people following curative intent treatment for non-small cell lung cancer: a randomized controlled trial	Cavalheri et al. (2017) [25]	17 individuals, mean age of 67 years (Standard Deviation: 9 years). Participants 6–10 weeks post-lobectomy for NSCLC or 4–8 weeks post-adjuvant chemotherapy	RCT to investigate the effects of supervised exercise training on multiple health outcomes in individuals who had completed curative intent treatment for NSCLC. - Intervention group: Participated in supervised exercise sessions twice weekly for 8 weeks. - Control: received usual care with no structured exercise intervention.	Outcomes measured included anxiety and depression levels, exercise capacity (assessed via peak oxygen consumption [VO_2_peak] and the 6MWD), physical activity and sedentary behavior, peripheral muscle strength (quadriceps and handgrip), HRQoL, fatigue, and lung function.	An 8-week supervised exercise training program led to improvements in exercise capacity. However, it did not lead to significant changes in physical activity levels, fatigue, anxiety, depression, or lung function. These results indicate that, while supervised exercise may enhance physical performance, its impact on broader health outcomes in this population appears to be limited.
Aerobic and resistance exercise improves physical fitness, bone health, and quality of life in overweight and obese breast cancer survivors: a randomized controlled trial	Dieli-Conwright et al. (2018) [26]	160 overweight and obese breast cancer survivors (female, ages 25–70 years)	RCT A 16-week supervised aerobic and resistance exercise program, three times a week.	FACT-B, SF-36, BFI, CES-D, Physical fitness tests (e.g., aerobic capacity, strength), bone health assessments (e.g., bone mineral density).	Participants in both exercise groups reported improvements in physical and emotional well-being. Notably, the exercise group showed a significant reduction in depression levels compared to the control group, with a between-group difference of 14.7 points (95% CI: 18.2, 9.7; *p* < 0.001).
Effects of dietary and physical activity interventions on generic and cancer-specific health-related quality of life, anxiety, and depression in colorectal cancer survivors: a randomized controlled trial	Ho et al. (2020) [27]	118 colorectal cancer survivors aged 18–80 years, with a mean age of 62 years. The inclusion criteria included having completed cancer treatment and having no significant comorbidities.	RCT with a two-group design. - Intervention group: received a combined program consisting of dietary modifications and physical activity, which included aerobic exercise, resistance training, and flexibility exercises. The intervention was delivered over a 6-month period through bi-weekly group sessions. - Control group: received standard care and did not participate in the dietary or physical activity components.	- Anxiety and Depression: measured by the HADS. - Physical Activity Levels: assessed through self-reported questionnaires and pedometer steps.	The combined dietary and physical activity intervention effectively reduced anxiety and depression while increasing physical activity in colorectal cancer survivors. These results indicate that lifestyle interventions can significantly improve both physical and mental health outcomes in this population. Participants in the intervention group showed significantly lower levels of anxiety and depression compared to those in the control group.
Motor Exercises Effect on Improving Shoulder Functioning, Functional Ability, Quality of Life, Depression and Anxiety For Women With Breast Cancer	Charati et al. (2022) [28]	70 women with breast cancer.	RCT. The participants were divided into intervention group that performed motor exercises for five weeks (n = 35) and control (n = 35) groups.	Depression and Anxiety: Measured using the HADS at baseline and five weeks post-surgery.	Significant reductions in depression and anxiety levels were observed in the intervention group compared to control (*p* < 0.05). In conclusion motor exercises effectively alleviated symptoms of anxiety and depression, thereby improving overall quality of life.

Abbreviations: Randomized controlled trial (RCT); Health-related quality of life (HRQoL); European Organisation for Research and Treatment of Cancer-Quality of Life Questionnaire Core 30 (EORT-QLQ-C30); Beck Depression Inventory (BDI); Non-small cell lung cancer (NSCLC); Six-minute Walk distance (6MWD); Functional Assessment of Cancer Therapy-Breast (FACT-B); Short Form-36 Health Survey (SF-36); Brief Fatigue Inventory (BFI); Center for Epidemiologic Studies Depression Scale (CES-D); Hospital Anxiety and Depression Scale (HADS).

**Table 2:** The modified version of Jadad Scale.

Authors	Was the Treatment Randomly Allocated?	Was the Randomization Procedure Described and Was Appropriate?	Was There a Description of Withdrawals and Dropout?	Was There a Clear Description of the Inclusion/Exclusion Criteria?	Were the Methods of Statistical Analysis Described?	Jadad score (0–5)
Aydin et al. (2021) [23]	Yes	No	Yes	Yes	Yes	4
Mikkelsen et al. (2022) [24]	Yes	Yes	Yes	Yes	Yes	5
Cavalheri et al. (2017) [25]	Yes	Yes	Yes	Yes	No	4
Dieli-Conwright et al. (2018) [26]	Yes	Yes	Ye	Yes	Yes	5
Ho et al. (2020) [27]	Yes	No	Yes	Yes	No	3
Charati et al. (2022) [28]	Yes	No	Yes	Yes	No	3

## Data Availability

The original contributions presented in the study are included in the article, and further inquiries can be directed to the corresponding author.

## Supplementary Materials

The following supporting information can be downloaded at: https://www.mdpi.com/article/10.3390/clinpract15100180/s1, The PRISMA 2020 checklist.

## Abbreviations

The following abbreviations are used in this manuscript:

RCTs	Randomized controlled trials
DALYs	Disability-adjusted life years
CRCI	cancer-related cognitive impairment
PRISMA	Preferred Reporting Items for Systematic Reviews and Meta-Analysis
MeSH	Medical Subject Headings
BDI	Beck Depression Inventory
NSCLC	Non-small cell lung cancer
6MWD	Six-minute Walk distance
HRQoL	Health-related quality of life
FACT-B	Functional Assessment of Cancer Therapy-Breast
SF-36	Short Form-36 Health Survey
BFI	Brief Fatigue Inventory
CES-D	Center for Epidemiologic Studies Depression Scale
HADS	Hospital Anxiety and Depression Scale
QoL	Quality of Life
